# Synthetic microneurotrophins in therapeutics of neurodegeneration

**DOI:** 10.18632/oncotarget.14667

**Published:** 2017-01-14

**Authors:** Achille Gravanis, Iosif Pediaditakis, Ioannis Charalampopoulos

**Affiliations:** Department of Pharmacology, Medical School University of Crete, Institute of Molecular Biology & Biotechnology, Foundation of Research & Technology- Hellas, Heraklion, Greece

**Keywords:** neurotrophin receptors, neuroinflammation, agonists, neurodegeneration, apoptosis, Neuroscience

Polypeptidic neurotrophins (such as the nerve growth factor (NGF), brain-derived neurotrophic factor (BDNF) and neurotrophin-3 (NT-3)), produced mainly in the brain, hold a central role in the control of neuronal development, survival, regeneration and plasticity [1]. Additionally, neurotrophins affect brain function, regulating axonal growth and dendritic sprouting and arborization, and synapse formation. They exert their multiple neurotrophic and neuroprotective actions through binding to specific pro-survival Trk (tyrosine kinase) receptors. Additionally, all neurotrophins are recognized by the pan-neurotrophin death receptor, p75^NTR^, which belongs to the TNF receptor superfamily.

Apoptotic neuronal loss is the common pathophysiological end-point of all neurodegenerative diseases and a large number of experimental and clinical studies implicate neurotrophins in this process [1]. It is now well documented that neurotrophin production declines in the degenerating brain, apparently leaving neuronal cells unprotected against pro-apoptotic insults. Although there are currently scarce effective therapeutic treatments for neurodegenerative diseases, apoptotic loss of neurons remains one of the major therapeutic targets. The effectiveness of neurotrophins in controlling neuronal apoptosis in various experimental animal models of neurodegenerative conditions has not yet been translated to clinical use, which has been hampered by their inability to pass the blood-brain-barrier (BBB) and their unstable serum pharmacokinetics and bioavailability.

During the last decade, our group described the molecular mechanism by which the endogenous neurosteroid dehydroepiandrosterone (DHEA), produced within the brain, protects neurons against apoptosis [2]. Surprisingly, DHEA was shown to bind and activate all Trk and p75^NTR^ neurotrophin receptors in various neuronal cell types. Based on these findings, we previously suggested that DHEA may have served as a primordial neurotrophic factor, promoting neuronal survival in the ancient less complex nervous systems. [3, 4]. The potential clinical use, however, of DHEA, as a long-term neuroprotective therapeutic is compromised by its multiple secondary effects via its binding to various steroid and neurotransmitter receptors and its central role as a precursor steroid in the biosynthesis of androgens and estrogens [5].

Our group has recently synthesized and screened a large chemical library of 17-carbon derivatives of DHEA for their ability to protect neurons against apoptosis as well as for their affinity for neurotrophin receptors [6]. BNN27, a BBB-permeable, C17-spiroepoxy steroid derivative, was shown to specifically interact with and activate the TrkA receptor of NGF, inducing phosphorylation of TrkA tyrosine residues and down-stream neuronal survival-related kinase signaling. BNN27 showed no affinity for TrkB, TrkC or steroid hormone receptors. Interestingly, “microneurotrophin” BNN27 potentiated the efficacy of low levels of NGF, by facilitating its binding to TrkA receptors and differentially inducing the fast return of internalized TrkA receptors into neuronal cell membranes. Furthermore, BNN27 synergized with low levels of NGF in promoting axonal outgrowth, and effectively rescued NGF-dependent and TrkA positive sympathetic and sensory neurons from apoptosis, *in vitro*, *ex vivo* and *in vivo* in NGF-null mice. Remarkably, BNN27 does not show the hyperalgesic effects of NGF [7] and has negligible toxicity in mice and rats. BNN27 also interacts with pan-neurotrophin p75^NTR^ receptors, in specific amino-residues of its extracellular domain, inducing the recruitment of p75^NTR^ receptor to its effector proteins RIP2 and TRAF6 and the simultaneous release of RhoGDI in primary neuronal cells.

**Figure 1:**
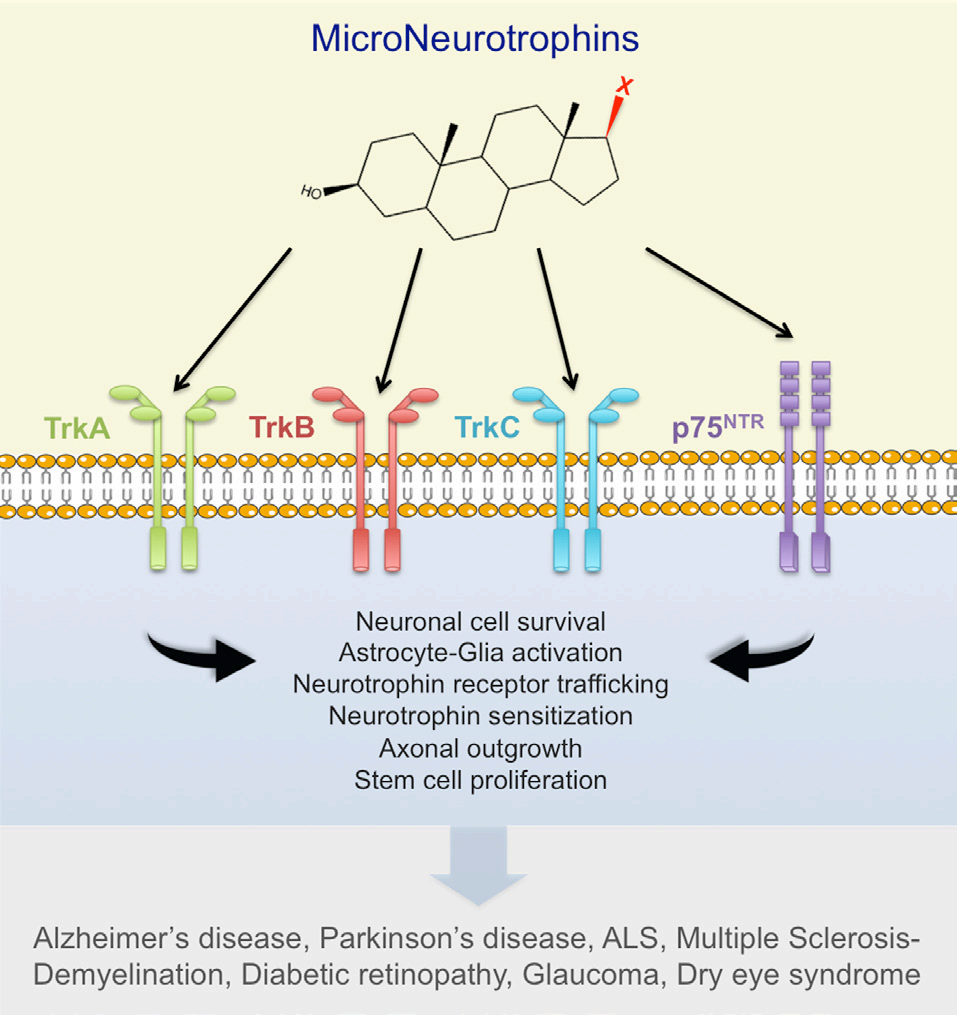
Blood-brain-barrier permeable 17-spiro-steroid derivatives bind to and activate neurotrophin receptors, protecting various neurons against apoptosis, *in vitro*, *ex vivo* and *in vivo* in animal models of neurodegenerative diseases. These synthetic microneurotrophins offer a new pharmacological tool to develop non-toxic, blood-brain-barrier permeable neurotrophin receptor agonists and antagonists, potentially effective in the treatment of neurodegenerative diseases, brain trauma and neuropathic pain.

The remarkable efficacy of BNN27 in protecting neurons from apoptosis is now being tested in various experimental animal models of neurodegenerative diseases, involving TrkA and p75^NTR^ receptors. Studies in the 5xFAD transgenic mice (Alzheimer’s disease), the cuprizone-treated and the experimental allergic encephalomyelitis mice (demyelination, multiple sclerosis) show that BNN27 and other synthetic “microneurotrophins” have neuroprotective actions in vivo. Systematic administration or local application with eye drops of BNN27 can also effectively mimic the beneficial effects of NGF against retina degeneration in the streptozotocin-induced diabetic rats, as well as in retina detachment in mice.

Taken together, the data are consistent with the suggestion that small, lipophilic molecules, like BNN27, able to interact with specific neurotrophin receptors may well represent novel lead compounds for developing non-toxic, BBB-permeable, neurotrophin receptor agonists and antagonists for therapeutic applications in neurodegenerative diseases, brain trauma and neuropathic pain. Several other “microneurotrophins” are currently being tested for their affinity for TrkB receptors and for their effectiveness in mimicking BDNF.
